# Association between constipation and the urine albumin-to-creatinine ratio in adults: the NHANES 2009–2010

**DOI:** 10.3389/fnut.2025.1477148

**Published:** 2025-02-17

**Authors:** Yuying Yang, Siyi Rao, Yongjie Zhuo, Yuan Fang, Jianxin Wan, Danyu You

**Affiliations:** ^1^Department of Nephrology, Blood Purification Research Center, The First Affiliated Hospital of Fujian Medical University, Fuzhou, China; ^2^Fujian Clinical Research Center for Metabolic Chronic Kidney Disease, The First Affiliated Hospital of Fujian Medical University, Fuzhou, China; ^3^Department of Nephrology, National Regional Medical Center, Binhai Campus of the First Affiliated Hospital, Fujian Medical University, Fuzhou, China

**Keywords:** association, constipation, albumin-to-creatinine ratio, adult, NHANES

## Abstract

**Objective:**

This study aimed to analyze the association between constipation and the urine albumin-to-creatinine ratio (ACR) using data from the National Health and Nutrition Examination Survey (NHANES) 2009–2010.

**Methods:**

In this cross-sectional study, a sample of 4,282 adults aged 20 and older was selected from the NHANES 2009–2010. Constipation was defined as having fewer than three bowel movements per week. The average of the two ACR measurements was used as the outcome variable. Logistic regression models (non-adjusted and multivariate adjusted models) were used to examine the relationship between constipation and ACR. Subgroup and interaction analyses related to gender, age, smoking, alcohol consumption, body mass index (BMI), hypertension, and diabetes were also conducted to assess the stability of the association between constipation and ACR.

**Results:**

In this study population of 4,282 individuals, 352 individuals with an ACR of 30 mg/g or higher were considered to have albuminuria. The prevalence of constipation was higher in the albuminuria group compared to the non-albuminuric group (6.4% vs. 3.5%, *p* = 0.002). The unadjusted model (Model I) showed an increased risk of ACR associated with constipation (OR 1.81, 95% CI 1.13–2.91, *p* = 0.014). After controlling for gender, age, race/ethnicity, marital status, and education level in Model II, the association between constipation and ACR remained significant (OR 2.20, 95% CI 1.34–3.60, *p* = 0.002). Upon further adjustment for BMI, smoking status, alcohol consumption, diabetes, hypertension, arthritis, asthma, coronary heart disease, liver disease, cancer, blood urea nitrogen (BUN), serum creatinine (Scr), uric acid (UA) and estimated glomerular filtration rate (eGFR) in Model III, the positive association between constipation and ACR was still significant (OR 1.88, 95% CI 1.09–3.23, *p* = 0.023). Subgroup analyses, stratified by gender, age, smoking status, alcohol consumption, BMI, hypertension, and diabetes, showed no statistically significant interactions (*p* > 0.05).

**Conclusion:**

In summary, this study found a positive association between constipation and urinary albumin excretion rate. The significant association between constipation and ACR highlights the need for clinicians to monitor urinary albumin levels in patients with constipation.

## Introduction

Albuminuria holds a significant role in the diagnosis, risk assessment, and management of chronic kidney disease (CKD) (1, 2). CKD is defined by the Kidney Disease: Improving Global Outcomes (KDIGO) guideline as abnormalities of kidney structure or function, present for a minimum of 3 months, with implications for health (2). Albuminuria, serving as a critical marker for kidney injury, can be evaluated through the urine albumin-to-creatinine ratio (ACR). An ACR of 30 mg/g or higher sustained over a period of at least three months indicates kidney damage (2). Beyond its role in diagnosing CKD and assessing its progression risk, the ACR is also used for the early evaluation of diabetic nephropathy and for guiding the administration of renin-angiotensin system (RAS) inhibitors in diabetic patients (3). Thus, the ACR plays a pivotal role in nephrology.

Constipation manifests with fewer than three bowel movements per week and/or more than one-quarter of bowel movements characterized by Bristol stool form types 1 or 2 (4). It is categorized into organic and functional types. Functional constipation is primarily attributed to lifestyle factors such as irregular bowel habits, dietary choices, and psychological stress, whereas organic constipation arises from structural diseases affecting the rectum and anus. The prevalence of constipation spans across all demographic groups, ranging from 2 to 27% (5). Notably, the incidence escalates with age, particularly affecting older adults women and pregnant women who are considered high-risk populations (4).

Constipation is common among patients with chronic kidney disease (CKD). Its causes include polypharmacy, low intake of dietary fiber, restricted fluid intake, lack of physical activity, changes in gut microbiota, and reduced gastrointestinal motility. Constipation has a negative impact on overall health, especially as its presence is associated with the deterioration of kidney function and an increased risk of developing end-stage renal disease (6). A study from the UK Biobank showed that constipation was independently associated with incident CKD in a large population-based longitudinal cohort, highlighting constipation as a potential risk factor or predictor of CKD development (7). A cohort study involving 3,504,732 US veterans with an eGFR of ≥60 mL/min per 1.73 m^2^ showed that patients with constipation had a higher incidence of CKD and end-stage renal disease (ESRD), and a faster decline in eGFR compared to those without constipation (8). The above studies indicate that constipation is such a significant health issue for patients with CKD, and improving constipation may help prevent or delay the progression of CKD. However, no studies have yet explored the relationship between constipation and ACR, which is a monitoring marker of early kidney damage in CKD. This study aims to analyze the association between constipation and ACR using data from the NHANES 2009–2010.

## Methods

### Data source

The National Health and Nutrition Examination Survey (NHANES)，a major program of the National Center for Health Statistics (NCHS), is a research program designed to assess the health and nutritional status of adults and children in the United States (9). This cross-sectional survey is unique in that it combines interviews and physical examinations. The NHANES interview includes demographic, socioeconomic, dietary, and health-related questions. The examination component encompasses medical, dental, and physiological measurements, as well as laboratory tests conducted by trained medical personnel.

**Figure 1:**
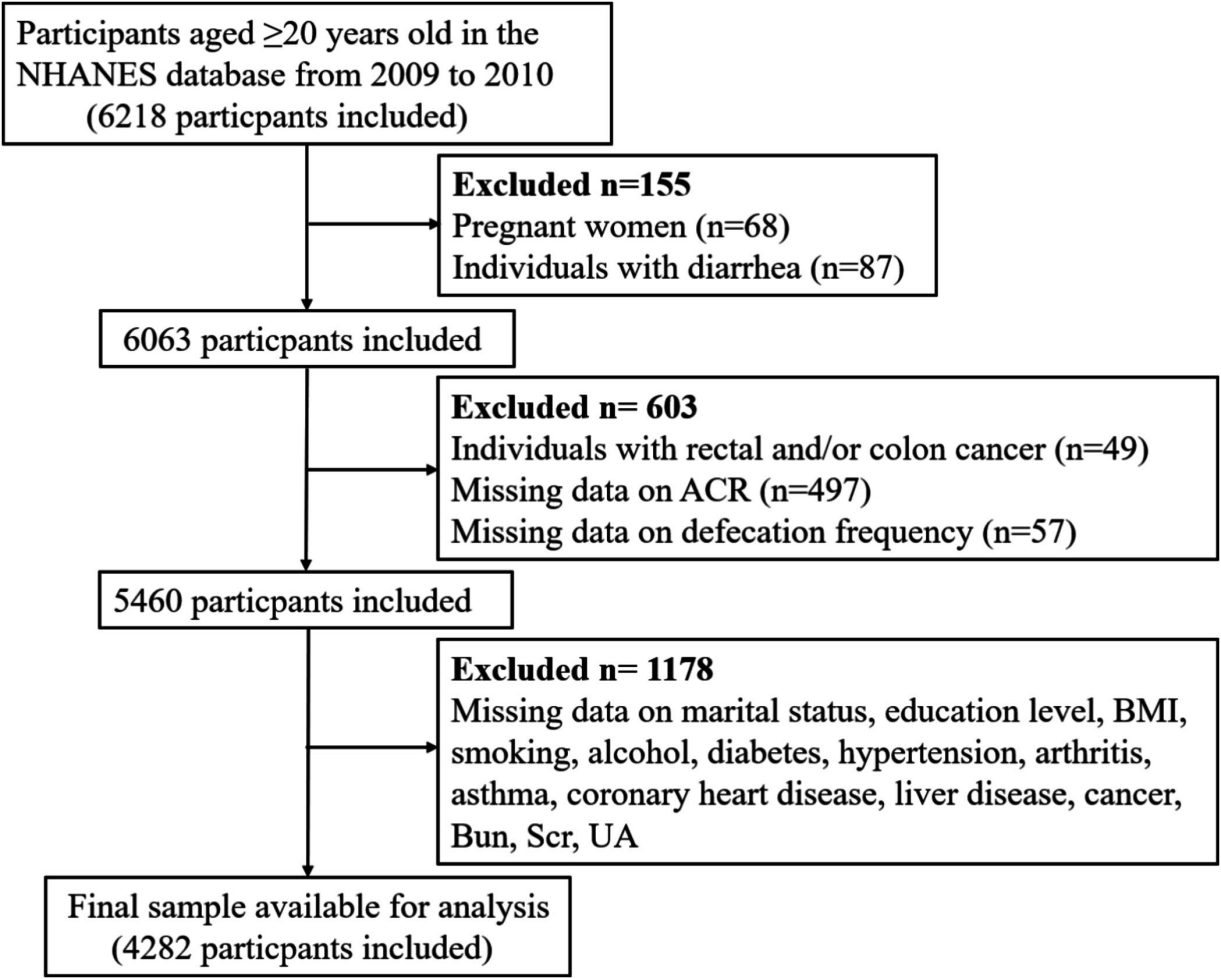
Flow diagram of the sample selection from the NHANES 2009–2010. NHANES: National Health and Nutrition Examination Survey, ACR: albumin-to-creatinine ratio, BMI, body mass index, BUN, blood urea nitrogen, Scr, serum creatinine; UA, uric acid; eGFR, estimated glomerular filtration rate.

Given the constipation data were only available in the NHANES 2005–2010, and the fact that the ACR measurements were obtained twice specifically in the NHANES 2009–2010, we believed that two measurements provided more accurate data than a single measurement. Therefore, we selected the NHANES 2009–2010 for our analysis, using the mean of the two ACR measurements for our study. Participants who were under 20 years of age, pregnant, diagnosed with rectal and/or colon cancer, experiencing diarrhea, and those with incomplete data across all variables were excluded. Consequently, 4,282 individuals were included in this analysis. Figure 1 provided a flow diagram illustrating this methodology.

### Constipation

The Bowel Health section of the mobile exam center (MEC) interview provided personal interview data on defecating function for adults aged 20 years and older. The NHANES employed both defecation frequency and stool consistency as criteria for measuring constipation among participants who completed the bowel health questionnaire. Drawing from the NHANES data, defecation frequency was the primary metric for identifying constipation, given the low association between stool frequency and consistency (10). Survey participants were asked to provide an estimate of their bowel movement frequency on a weekly basis. According to the responses, fewer than 3 bowel movements per week were categorized as constipation, 3 to 21 bowel movements per week were considered normal, and more than 21 bowel movements per week were identified as diarrhea (11, 12).

### ACR

Urinary albumin and creatinine were measured using a modified Jaffe kinetic method and solid-phase fluorescent immunoassay applied to spot urine samples. They were measured in a random urine collected in the MEC (first collection) and a first morning void urine collected by the participant at home (second collection) (9). Urine albumin (mg/mL) and urinary creatinine (mg/dL) were converted to albumin-to-creatinine ratio (ACR) in mg/g. The average of these two ACR measurements was used in our study. ACR was considered the outcome variable in our analysis. The normal group (non-albuminuric group) was defined as having an ACR less than 30 mg/g, while the albuminuria group was defined as having an ACR greater than 30 mg/g (2).

### Covariates

Several covariates were evaluated as potential factors associated with ACR (13, 14). The covariates considered in this study included gender, age, race/ethnicity, marital status, education level, body mass index (BMI), smoking, alcohol consumption, diabetes, hypertension, arthritis, asthma, coronary heart disease, liver disease, cancer, blood urea nitrogen (BUN), serum creatinine (Scr), uric acid (UA) and estimated glomerular filtration rate (eGFR). The reasons for selecting BMI, smoking, alcohol consumption, diabetes, hypertension, arthritis, asthma, coronary heart disease, liver disease, and cancer as covariates are that they are related to constipation and/or ACR (14–16). Race/ethnicity was categorized into five groups: Mexican American, other Hispanic, Non-Hispanic White, Non-Hispanic Black, and other races. Marital status had three categories: married or living together; divorced, separated, or widowed; and never married. Education level was divided into two groups: “≤ high school” and “> high school.” BMI was classified into two groups: under/normal weight (< 25.0 kg/m^2^) and overweight/obese (≥ 25 kg/m^2^). A smoker was defined as someone who had smoked at least 100 cigarettes in their lifetime. A drinker was defined as a person who consumed at least 12 drinks annually. Medical comorbidities were also included based on the Medical Conditions Questionnaire. The comorbid conditions considered were diabetes, hypertension, arthritis, asthma, coronary heart disease, liver disease, and cancer. BUN, Scr, and UA data were obtained from the Laboratory Data-Standard Biochemistry Profile (BIOPRO_F). The eGFR was calculated using the CKD-EPI formula (17).

### Statistical analyses

A descriptive analysis was performed on all participants. Continuous data from all participants were analyzed using the mean and standard deviation (SD). Categorical variables were represented by percentages (%). An analysis of categorical variables was conducted using the Chi-square test. Continuous variables, such as age, were analyzed using a T-test. Logistic regression models were used to examine the relationship between constipation and ACR. Both non-adjusted and multivariate adjusted models were used: Model I, without adjustment for any covariates; Model II, adjusted for gender, age, race/ethnicity, marital status, and education level; Model III, adjusted for the covariates in Model II as well as BMI, smoking, alcohol, diabetes, hypertension, arthritis, asthma, coronary heart disease, liver disease, cancer, BUN, Scr, UA and eGFR. Subgroup and interaction analyses related to gender, age, smoking, alcohol consumption, BMI, hypertension, and diabetes were also conducted to assess the stability of the association between constipation and ACR. Statistical significance was determined by comparing the adjusted odds ratios (ORs) to 1.0 and describing 95% confidence intervals (CIs).

All statistical analyses were conducted using SPSS 25.0, R (version 3.4.3), EmpowerStats XYS. A two-tailed test was performed, and a *p*-value of less than 0.05 was considered statistically significant.

## Results

### Subject characteristics

Table 1 illustrates a comparison of the clinical characteristics between participants with albuminuria and those without. In this study population of 4,282 individuals, 352 individuals with an ACR of 30 mg/g or higher were considered to have albuminuria. Participants with albuminuria were more likely to be older, divorced, separated, or widowed, to have lower levels of education, to have a higher BMI (BMI ≥ 25), and to be smokers and non-drinkers. Among those with comorbidities, the albuminuria group was more likely to be associated with diabetes, hypertension, arthritis, coronary heart disease, liver disease and cancer. Biochemical tests showed that the albuminuria group had higher levels of BUN, Scr and UA. The albuminuria group also had lower level of eGFR calculated by CKD-EPI formula. The prevalence of constipation was higher in the albuminuria group compared to the non-albuminuric group (6.4% vs. 3.5%, *p* = 0.002).

**Table 1:** Characteristics of participants in the normal group (non-albuminuric group) and the albuminuria group.

Variables	Normal group (ACR < 30 mg/g)	Albuminuria group (ACR ≥ 30 mg/g)	*p-*value
	*n* = 3,930	*n* = 352	
Age (years, mean ± SD)	49.2 ± 17.1	60.6 ± 16.1	**<0.001**
Gender, *n* (%)			0.201
Male	1948 (49.6%)	187 (53.1%)	
Female	1982 (50.4%)	165 (46.9%)	
Race/ Ethnicity, *n* (%)			**0.045**
Mexican American	685 (17.4%)	79 (22.4%)	
Other Hispanic	400 (10.2%)	40 (11.4%)	
Non-Hispanic White	2005 (51.0%)	152 (43.2%)	
Non-Hispanic Black	648 (16.5%)	65 (18.5%)	
Other Race -Including Multi-Racial	192 (4.9%)	16 (4.5%)	
Marital Status, *n* (%)			**0.028**
Married or living with partner	2,443 (62.2%)	220 (62.5%)	
Divorced, separated, or widowed	858 (21.8%)	92 (26.1%)	
Never married	629 (16.0%)	40 (11.4%)	
Education Level, *n* (%)			**<0.001**
≤ high school	1918 (48.8%)	215 (61.1%)	
> high school	2012 (51.2%)	137 (38.9%)	
BMI, *n* (%)			**0.036**
<25	1,086 (27.6%)	79 (22.4%)	
≥25	2,844 (72.4%)	273 (77.6%)	
Smoking, *n* (%)			**<0.001**
No	2,144 (54.6%)	158 (44.9%)	
Yes	1786 (45.4%)	194 (55.1%)	
Alcohol, *n* (%)			**0.002**
No	1,012 (25.8%)	117 (33.2%)	
Yes	2,918 (74.2%)	235 (66.8%)	
Diabetes, *n* (%)			**<0.001**
No	3,564 (90.7%)	236 (67.0%)	
Yes	366 (9.3%)	116 (33.0%)	
Hypertension, *n* (%)			**<0.001**
No	2,640 (67.2%)	133 (37.8%)	
Yes	1,290 (32.8%)	219 (62.2%)	
Arthritis, *n* (%)			**<0.001**
No	2,894 (73.6%)	195 (55.4%)	
Yes	1,036 (26.4%)	157 (44.6%)	
Asthma, *n* (%)			0.390
No	3,397 (86.4%)	310 (88.1%)	
Yes	533 (13.6%)	42 (11.9%)	
Coronary heart disease, *n* (%)			**<0.001**
No	3,789 (96.4%)	317 (90.1%)	
Yes	141 (3.6%)	35 (9.9%)	
Liver disease, *n* (%)			**<0.001**
No	3,819 (97.2%)	330 (93.8%)	
Yes	111 (2.8%)	22 (6.2%)	
Cancer, *n* (%)			**<0.001**
No	3,571 (90.9%)	299 (84.9%)	
Yes	359 (9.1%)	53 (15.1%)	
BUN (mmol/L, mean ± SD)	4.7 ± 1.9	6.2 ± 3.4	**<0.001**
Scr (μmol/L, mean ± SD)	77.5 ± 20.2	104.6 ± 96.2	**<0.001**
UA(μmol/L, mean ± SD)	324.1 ± 85.9	350.3 ± 97.3	**<0.001**
eGFR	115.3 ± 44.8	95.1 ± 54.3	**<0.001**
Constipation, *n* (%)			**0.013**
No	3,797 (96.6%)	331 (94.0%)	
Yes	133 (3.4%)	21 (6.0%)	

Continuous data were analyzed using the mean and standard deviation (SD). Categorical variables were represented by percentages (%). ACR, albumin-to-creatinine ratio, BMI: body mass index, BUN: blood urea nitrogen, Scr, serum creatinine, UA, uric acid; eGFR, estimated glomerular filtration rate. Statistically significant results (*p*-values < 0.05) are indicated in bold.

### Association between constipation and ACR

**Figure 2:**
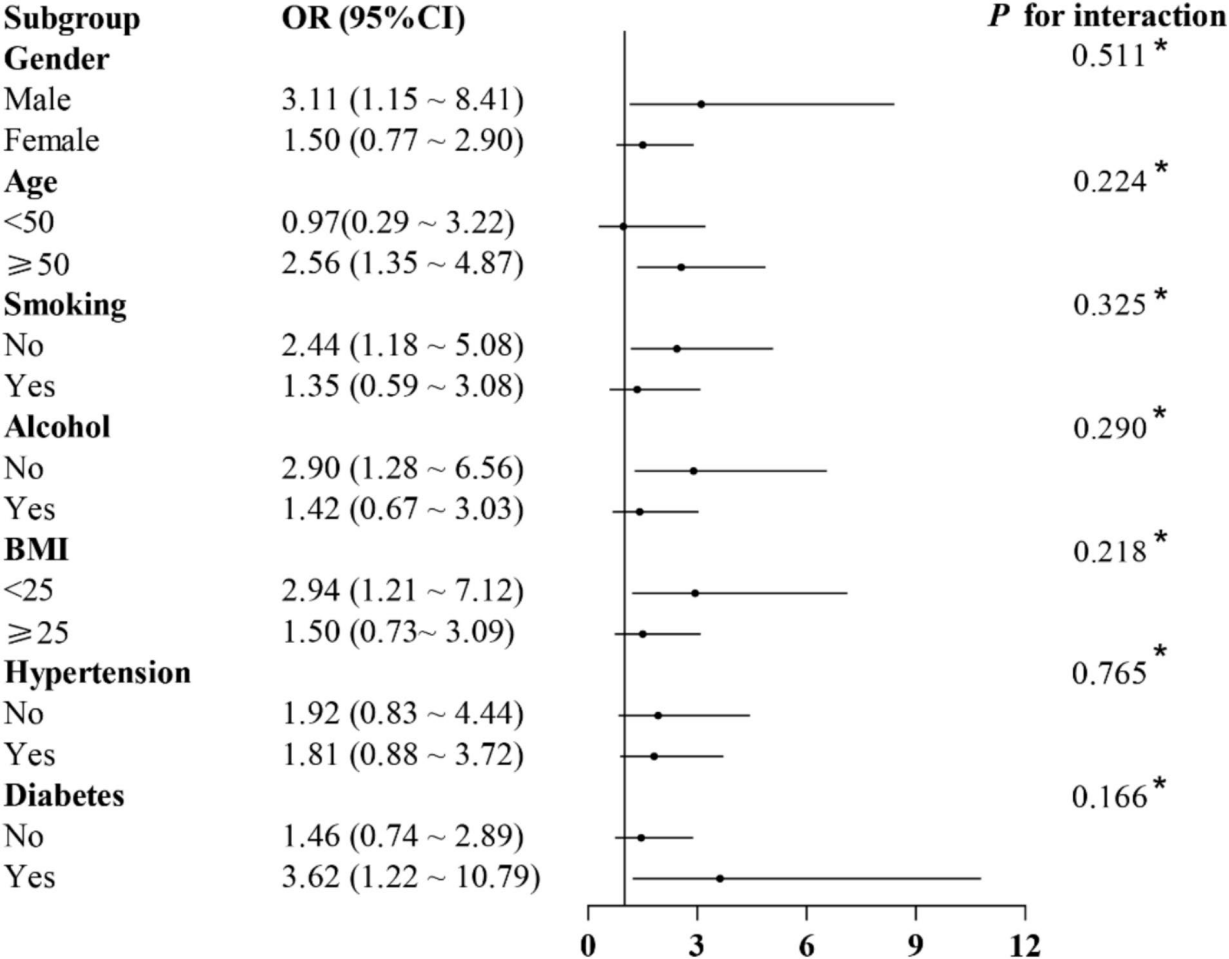
Association between constipation and ACR in different subgroups. Adjusted for gender, age, race/ethnicity, marital status, education level, BMI, smoking status, alcohol consumption, diabetes, hypertension, arthritis, asthma, coronary heart disease, liver disease, cancer, BUN, SCR, UA and eGFR.

Table 2 presents the results of the logistic regression analysis showing the association between constipation and ACR. The unadjusted model (Model I) showed an increased risk of ACR associated with constipation (OR: 1.81). After controlling for gender, age, race/ethnicity, marital status, and education level in Model II, the association between constipation and ACR remained significant (OR: 2.20). Upon further adjustment for BMI, smoking status, alcohol consumption, diabetes, hypertension, arthritis, asthma, coronary heart disease, liver disease, cancer, BUN, SCR, UA and eGFR in Model III, the positive association between the two was still significant (OR: 1.88). Figure 2 shows subgroup analyses stratified by gender, age, smoking status, alcohol consumption, BMI, hypertension, and diabetes, did not reveal any statistically significant interactions (*p* > 0.05) when adjusted for gender, age, race/ethnicity, marital status, education level, BMI, smoking status, alcohol consumption, diabetes, hypertension, arthritis, asthma, coronary heart disease, liver disease, cancer, BUN, SCR, UA and eGFR. We found that the association between constipation and ACR was relatively stable in every subgroup.

**Table 2:** Multivariate regression analysis of the association between constipation and ACR.

Exposure	Model I OR, (95%CI), *p*	Model II OR, (95%CI), *p*	Model III OR, (95%CI), *p*
Constipation	1.81 (1.13–2.91), **0.014**	2.20(1.34–3.60), **0.002**	1.88 (1.09–3.23), **0.023**

Model I: no adjusted.

Model II: adjusted for gender, age, race/ethnicity, marital status, and education level.

Model III: Model II and adjusted for BMI, smoking, alcohol, diabetes, hypertension, arthritis, asthma, coronary heart disease, liver disease, cancer, BUN, Scr, UA and eGFR. Statistically significant results (*p*-values < 0.05) are indicated in bold.

## Discussion

Based on the NHANES, a nationwide survey in the United States, we found that individuals with constipation had a higher risk of developing ACR compared to those without constipation. Subgroup analyses, stratified by gender, age, smoking status, alcohol consumption, BMI, hypertension, and diabetes, revealed that the exposure factor of constipation did not exhibit significant interactions with these subgroup factors, suggesting that the association between constipation and ACR was not influenced by various subgroup factors. This finding may suggest that the relationship between constipation and ACR could be applied to early kidney damage monitoring or to further advance the gut-kidney axis theory. Integrating albuminuria monitoring into the standard evaluation of chronic constipation patients may enhance the detection of early kidney damage.

Over the past 20 years, CKD has become a major public health concern worldwide. In 2017, approximately 697.5 million people globally were affected by CKD, accounting for 9.1% of the world’s population, with about one-third of the patients coming from China and India (18). CKD can lead to an increased incidence of cardiovascular events, which are the leading cause of death globally. Epidemiological data from 2017 showed that cardiovascular diseases caused by impaired kidney function resulted in 1.4 million deaths, accounting for 7.6% of all cardiovascular disease deaths (18). ACR is an important basis for the diagnosis, staging, progression, and risk assessment of CKD. The KDIGO guidelines suggest dividing ACR into three categories: A1, less than 30 mg/g (less than 3 mg/mmol); A2, 30 to 300 mg/g (3 to 30 mg/mmol); A3, greater than 300 mg/g (greater than 30 mg/mmol). Among them, an ACR of 30 mg/g or more (3 mg/mmol or more) is considered a sign of kidney damage, with the range of 30 to 300 mg/g (3 to 30 mg/mmol) referred to as microalbuminuria, and an ACR greater than 300 mg/g (greater than 30 mg/mmol) is referred to as macroalbuminuria. The risk of CKD progression increases with the increase of ACR (2, 19). Therefore, our study opted to define a ACR of 30 mg/g or greater as the albuminuria group. Additionally, microalbuminuria is also considered the earliest sign of diabetic nephropathy. To effectively control diabetic nephropathy and its consequences at an early stage, it is crucial to detect microalbuminuria as soon as possible (20). Moreover, ACR is also used as an important monitoring indicator for the efficacy of diabetic nephropathy medications, such as RAS inhibitors and sodium-glucose cotransporter 2 (SGLT2) inhibitors (3). Given the significant role of ACR in kidney disease, it is particularly important to identify risk factors associated with it in the general population.

Constipation is a common symptom of functional gastrointestinal disorders and is one of the most common diseases encountered in daily clinical practice. In the general population, about 30% of people will experience constipation problems in their lifetime, with the older adults and women being the most affected (4). It has been reported that the prevalence of constipation in patients with CKD, especially those with advanced CKD, is higher than in the general population. For example, in a study comparing the prevalence of constipation in patients undergoing continuous ambulatory peritoneal dialysis (CAPD) and hemodialysis (HD), researchers found that the prevalence of constipation was 28.9% in 204 CAPD patients and 63.1% in 268 HD patients; the relative risk of constipation in HD patients was 3.14 times that of CAPD patients (21). A systematic review including 30 studies (24 cross-sectional studies and 6 cohort studies) with 5,161 patients (3,804 HD and 1,507 PD) indicated that the most common gastrointestinal symptom in patients undergoing dialysis for end-stage renal disease (ESRD) is constipation, with a prevalence of 1.6 to 70.7% in HD patients and 14.2 to 90.3% in PD patients (22).

Patients with CKD may be more prone to constipation than the general population due to restrictions on water intake, lack of physical activity, and low intake of dietary fiber (6). CKD patients often need to take medications that can induce constipation due to their condition, such as iron supplements, antihypertensives, potassium-lowering agents, phosphate binders, and diuretics. Certain comorbidities (diabetes, hypercalcemia, etc.) are also likely to induce constipation (6). In addition, increased uremic toxins and changes in gut microbiota, both common in advanced CKD patients, are also associated with the high prevalence of constipation in CKD (23).

The prevalence of constipation is higher in patients with CKD, but it is currently unclear whether constipation can lead to CKD. In a nationwide cohort of 3,504,732 United States veterans with an eGFR ≥60 mL/min/1.73 m^2^, researchers analyzed the association between constipation status and severity and the incidence of CKD, end-stage renal disease (ESRD), and changes in eGFR using Cox models and multinomial logistic regression models. The average age of these patients was 60.0 ± 14.1 years; 93.2% of the patients were male, and 24.7% had diabetes. After adjusting for multiple variables, compared with patients without constipation, those with constipation had a higher incidence of CKD (HR: 1.13; 95% CI: 1.11 to 1.14) and ESRD (HR: 1.09; 95% CI: 1.01 to 1.18), and a faster decline in eGFR. The study confirmed that the status and severity of constipation were associated with a higher risk of incident CKD and ESRD, as well as a progressive decline in eGFR, independent of known risk factors (8). A study from the UK, which included 118,020 participants with general practice follow-up data, showed that after excluding individuals with pre-existing CKD or missing covariates, 6,833 patients (5.8%) developed CKD during a median follow-up of 7.4 years (7). In another study focusing on patients with CKD, researchers identified newly diagnosed CKD patients without a history of constipation from the Taiwan National Health Insurance database between 2000 and 2011. Among these individuals, some developed constipation later on (constipation group), while others did not (non-constipation group). The incidence and risk of ESRD by the end of 2013 in both groups were compared using Cox proportional hazards models. The results showed that the incidence rates of ESRD were 22.9 per 1,000 person-years for the constipation group and 12.2 for the non-constipation group; the adjusted hazard ratio was 1.90 (95% CI, 1.60–2.27). Therefore, the researchers concluded that among newly diagnosed CKD patients, those with new-onset constipation, compared to those without constipation, had a higher risk of developing ESRD, and more severe constipation further increased this risk (24). These three studies—one with an older adults population and the other two with CKD populations—all had eGFR as the outcome variable and did not observe the association between constipation and albuminuria. Whether constipation is related to albuminuria in the general adult population has not been reported. Our study is the first to use the NHANES database to observe the association between constipation and albuminuria in the general adult population. We defined the albuminuria group as individuals with an ACR ≥30 mg/g to investigate the relationship between constipation and albuminuria. Our findings indicated that in the general adult population, there was a significant association between constipation and ACR. Upon adjustment for various confounding factors such as diabetes, hypertension, smoking, renal function, and other comorbidities in Model III, the positive association between constipation and ACR was still significant (OR: 1.88, 95%CI: 1.09–3.23, *p* = 0.023). OR = 1.88, which indicated that the probability of ACR occurring in the people with constipation was 1.88 times the probability of ACR occurring in the people without constipation (Table 2).

Constipation is associated with albuminuria, which may be related to gut microbiota dysbiosis. It is well known that the gut microbiome is composed of various microorganisms, such as bacteria, viruses, protozoa, and fungi, which have a significant impact on the host during homeostasis and disease. The concept of the “gut-kidney axis, “proposed in recent years, emphasizes the impact of gut microbes and their metabolites on the kidneys, with dysbiosis being considered a triggering event for several diseases, including CKD (16). For example, rats with experimental membranous nephropathy exhibited gut microbiota dysbiosis, characterized by a higher Firmicutes/Bacteroidetes ratio and reduced diversity and richness of the microbiota. Treatment with coumarins isolated from *Hydrangea paniculata* (HP) reversed these changes. Fecal microbiota transplantation (FMT) of HP-treated feces into MN rats moderately reduced endotoxemia and albuminuria (25). Gut microbiota dysbiosis in constipated individuals is associated with an increased ACR, which may be related to factors such as inflammation and metabolism. Modulating the gut microbiota can alleviate renal inflammation in CKD mice via the NF-κB/TGF-*β* pathway, thereby reducing proteinuria and improving renal fibrosis (26). Gut microbiota dysbiosis leads to an increased production of acetate, which can disrupt cholesterol homeostasis and promote podocyte insulin resistance by activating GPR43, thereby causing albuminuria and renal tubular injury in rats with diabetic nephropathy (27, 28). Therefore, in the general population, improving constipation by modulating the gut microbiota may help prevent the risk of developing albuminuria in the future. Additionally, the current guidelines for chronic constipation do not explicitly mention the monitoring of albuminuria. Therefore, incorporating albuminuria monitoring into the routine assessment of chronic constipation patients can better detect early kidney damage.

However, our study has several limitations. Firstly, because the study is cross-sectional, it can only indicate an association between constipation and ACR, and cannot infer causality between the two, hence the possibility of a reverse causal relationship also exists. This limits the interpretation and application of the research results, and they cannot be directly used to make causal inferences and develop interventions. In the future, we will conduct prospective studies to better establish causality between constipation and albuminuria. Secondly, since only the data from 2009 to 2010 underwent two ACR tests and the data from 2005 to 2010 conducted a questionnaire survey on bowel health, we only selected the data from 2009–2010 for analysis, hence the sample size is relatively small. Since data collection was completed within a relatively short period of time, it was unable to capture the changing trends and dynamic processes of variables over time. This limitation means that it does not provide sufficient information and makes it difficult to meet the needs of in-depth research. Thirdly, even after controlling for potential confounding factors, observational studies are also susceptible to the influence of residual confounding factors. The presence of confounding factors can bias the research results, leading to misunderstandings and incorrect conclusions about the main research question.

## Conclusion

In summary, this study found a positive association between constipation and urinary albumin excretion rate. This positive association persisted even after adjusting for a multitude of covariates such as diabetes, hypertension, smoking, BMI, etc. The significant association between constipation and ACR highlights the need for clinicians to monitor urinary albumin levels in patients with constipation. This monitoring can aid in the early detection of kidney damage and facilitate timely intervention.

## Data Availability

Publicly available datasets were analyzed in this study. This data can be found here: the data sets generated and analyzed during the current study are available in the National Center for Health Statistics (NCHS), https://www.cdc.gov/nchs/nhanes/index.htm.

## Glossary

ACR: Albumin-to-creatinine ratio
BMI: Body mass index
BUN: Blood urea nitrogen
CAPD: Continuous ambulatory peritoneal dialysis
CIs: Confidence intervals
CKD: Chronic kidney disease
eGFR: Estimated glomerular filtration rate
ESRD: End-stage renal disease
FMT: Fecal microbiota transplantation
HD: Hemodialysis
HP: *Hydrangea paniculata*
KDIGO: Kidney Disease: Improving Global Outcomes
MEC: Mobile exam center
NHANES: National Health and Nutrition Examination Survey
NCHS: National Center for Health Statistics
RAS: Renin-angiotensin system
Scr: Serum creatinine
SD: Standard deviation
SGLT2: Sodium-glucose cotransporter 2
UA: Uric acid
